# Accelerated regeneration of the skeletal muscle in RNF13-knockout mice is mediated by macrophage-secreted IL-4/IL-6

**DOI:** 10.1007/s13238-014-0025-4

**Published:** 2014-02-22

**Authors:** Jiao Meng, Xiaoting Zou, Rimao Wu, Ran Zhong, Dahai Zhu, Yong Zhang

**Affiliations:** The National Laboratory of Medical Molecular Biology, Institute of Basic Medical Sciences, Chinese Academy of Medical Sciences and School of Basic Medicine, Peking Union Medical College, Beijing, 100005 China

**Keywords:** RNF13, muscle regeneration, satellite cell niche, IL-4/IL-6

## Abstract

RING finger protein 13 (RNF13) is a newly identified E3 ligase reported to be functionally significant in the regulation of cancer development, muscle cell growth, and neuronal development. In this study, the function of RNF13 in cardiotoxin-induced skeletal muscle regeneration was investigated using *RNF13*-knockout mice. *RNF13*^*-/-*^ mice exhibited enhanced muscle regeneration—characterized by accelerated satellite cell proliferation—compared with wild-type mice. The expression of RNF13 was remarkably induced in macrophages rather than in the satellite cells of wild-type mice at the very early stage of muscle damage. This result indicated that inflammatory cells are important in RNF13-mediated satellite cell functions. The cytokine levels in skeletal muscles were further analyzed and showed that *RNF13*^*-/-*^ mice produced greater amounts of various cytokines than wild-type mice. Among these, IL-4 and IL-6 levels significantly increased in *RNF*13^*-/-*^ mice. The accelerated muscle regeneration phenotype was abrogated by inhibiting IL-4/IL-6 action in *RNF13*^*-/-*^ mice with blocking antibodies. These results indicate that RNF13 deficiency promotes skeletal muscle regeneration via the effects on satellite cell niche mediated by IL-4 and IL-6.

**Electronic supplementary material:**

The online version of this article (doi:10.1007/s13238-014-0025-4) contains supplementary material, which is available to authorized users.

## Introduction

Regeneration of skeletal muscles after injury is a coordinated process that progresses through four phases: degeneration; inflammation; regeneration; and remodeling-repair (Carosio et al., 2009; Ten Broek et al., 2010). Injury of myofibers leads to rapid necrosis associated with sarcomere disruption, cell permeabilization, and proteolysis, which induces an acute inflammatory response characterized by the invasion of inflammatory cells (Brunelli and Rovere-Querini, 2008). The regeneration process is characterized by the activation of muscle stem cells (satellite cells), followed by myoblast differentiation and fusion into regenerated myofibers (Hawke, 2001). The final stages of muscle repair include the maturation of regenerated myofibers, the remodeling of extracellular matrix, and the recovery of the contractile capacity of injured muscles (Carosio et al., 2009).

Satellite cells, a quiescent population of myogenic cells that reside between the basal lamina and the plasmalemma, play the central role in skeletal muscle regeneration (Mauro, 1961). Satellite cells can be identified and isolated using a panel of satellite cell-associated markers, such as the paired-box transcription factor Pax7, and some cell surface markers, including M-cadherin, syndecan-3 and -4, CD34, α7-integrin, CXCR4 [chemokine (C-X-C motif) receptor 4], and SM/C2.6 antigen (Vcam) (Beauchamp et al., 2000; Cerletti et al., 2008; Cornelison et al., 2004; Fukada et al., 2007; Gnocchi et al., 2009; Kuang et al., 2008). Among these markers, Pax7 is specifically expressed in quiescent and activated satellite cells and is commonly used as a marker (Halevy et al., 2004; Relaix et al., 2005). MyoD expression, which is remarkably increased in activated satellite cells after injury, is widely regarded as the hallmark of activated satellite cells (Kanisicak et al., 2009; Zammit and Beauchamp, 2001; Zammit et al., 2002). In general, the immediate environment- or niche-of satellite cells participates in the modulation of satellite cell functions. Numerous factors in this niche influence satellite cell behavior, including adjacent myofibers, basal lamina, microvasculature, and neuromuscular junctions, as well as the secretion of multiple factors by interstitial cells and immune cells (Dhawan and Rando, 2005; Gopinath and Rando, 2008; Kuang et al., 2008; Ten Broek et al., 2010). Among these factors, inflammatory cells infiltrating the injured muscle perform a very important function in satellite cell activation, proliferation, and differentiation.

The innate immune system is activated as an immediate response to acute muscle injury. The first event in this process involves the invasion of damaged areas by inflammatory cells. Neutrophils, the first invasive inflammatory cells, infiltrate the damaged site in 2 h after injury. However, neutrophils die during inflammation by apoptosis and become undetectable in 3–4 days after injury (Tidball and Villalta, 2010). Neutrophils mainly function to promote the removal of cellular debris by secreting cytokines (Tidball, 2005). The next immune cells to invade are macrophages, which are the major cells involved in inflammation and regeneration. As macrophage function is suppressed, the proliferation and differentiation of muscle satellite cells are delayed *in vivo*; as a result, incomplete muscle regeneration and fibrosis occur (Segawa et al., 2008). In previous studies (Brunelli and Rovere-Querini, 2008; Chazaud et al., 2009; Kharraz et al., 2013), macrophages are a heterogeneous population involved in different functions at various stages after damage. Macrophages are classified as M1 and M2, corresponding to classical and alternative activation, respectively. Classically activated M1 macrophages, which are usually found at the early stage, produce high levels of pro-inflammatory cytokines. These macrophages mainly function to phagocytose necrotic fiber debris. M2 macrophages regulate the function of satellite cells by secreting cytokines and chemokines that facilitate vascular and muscle fiber repair. Thus, macrophages are the predominant inflammatory cells and local source of cytokines; playing important roles in the regulation of satellite cell activation, proliferation, and differentiation.

The function of cytokines and chemokines in regulating satellite cell function has been the focus of several studies. The expression and/or secretion of more than 100 cytokines increase by more than fivefold after toxin injection (Hirata et al., 2003). This altered expression and secretion pattern after injury suggests the important function of various cytokines in the regulation of satellite cell activation, proliferation, and differentiation. For instance, tumor necrosis factor (TNF)-α has been shown to increase with muscle injury and regulate muscle regeneration through activation of p38; conversely, TNF-α deficiency impairs muscle regeneration (Chen et al., 2005, 2007; Warren et al., 2002). The interleukin (IL)-6/STAT3 (signal transducer and activator of transcription 3) pathway is essential for macrophage infiltration and myoblast proliferation during muscle regeneration (Zhang et al., 2013a). IL-10 plays an important role in muscle regeneration by regulating the transformation of macrophages from an M1 to an M2 phenotype (Deng et al., 2012). Another study has demonstrated that the activation of IL-4/IL-13 signaling promotes the proliferation of fibro/adipocyte progenitors to support myogenesis while inhibiting their differentiation into adipocytes (Heredia et al., 2013). However, the specific function of these cytokines remains unclear. Under normal conditions, the repertoire and function of cytokines in the satellite cell niche vary, highlighting the importance of investigating the combined effects of cytokines.

We have previously demonstrated that the RING finger ubiquitin ligase RNF13 is a widely expressed membrane-associated E3 ligase evolutionarily conserved among many metazoans (Jin et al., 2011; Zhang et al., 2009; Zhang et al., 2010). RNF13 negatively regulates the proliferation of chicken myoblasts (Zhang et al., 2010). The expression of RNF13, which is positively regulated by myostatin, gradually decreases during skeletal myogenesis. The overexpression of RNF13 inhibits cell proliferation, an effect dependent on the E3 ligase activity of RNF13 (Zhang et al., 2010). We identified snapin, a SNAP25-interacting protein, as a substrate of RNF13, and showed that RNF13 was involved in the regulation of the soluble N-ethylmaleimide-sensitive fusion protein attachment protein receptor (SNARE) complex via snapin ubiquitination (Zhang et al., 2013b).

To further investigate the function of RNF13 in adult muscle development, we established a cardiotoxin (CTX)-induced muscle regeneration model in *RNF13*-knockout (*RNF13*^*-/-*^) mice (Zhang et al., 2013b). *RNF13*^*-/-*^ mice exhibited accelerated muscle regeneration after injury. Moreover, RNF13 was significantly induced in inflammatory cells as early as 1 h after CTX injection in wild-type mice. Importantly, RNF13 influenced the concentration of numerous cytokines in damaged areas and modulated muscle regeneration by affecting IL-4/IL-6 function. This study is the first to demonstrate that E3 ligase RNF13 regulates the function of satellite cells by modulating cytokine composition.

## Results

### Loss of *RNF13* accelerates skeletal muscle regeneration

**Figure 1:**
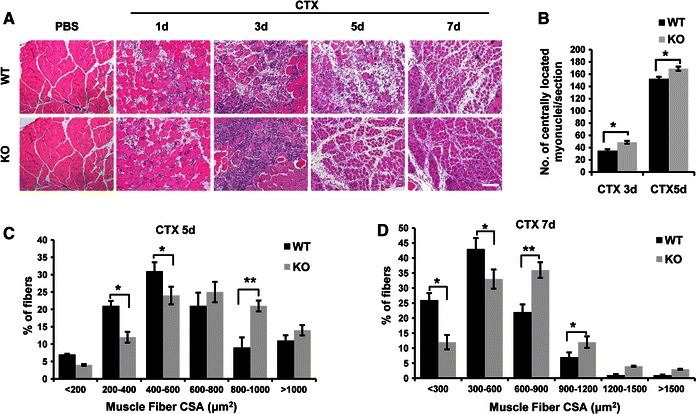
**Histological analysis of muscle regeneration in*RNF13***^**-/-**^**and*RNF13***^**+/+**^**mice**. (A) Sections from the TA muscles of *RNF13*^-/-^ and *RNF13*^+/+^ mice at different times (0, 1, 3, 5, and 7 d) after CTX injection were stained with H&E. Scale bar = 600 μm. (B) The percentage of regenerating myofibers, characterized by their centrally located myonuclei, was calculated in H&E-stained sections from *RNF13*^-/-^ and *RNF13*^+/+^ TA muscles at 3 d and 5 d post-injury. (C and D) The cross-sectional areas (CSAs) of regenerating muscle fibers 5 d and 7 d after injury were analyzed in H&E-stained sections. Five pairs of mice were used for each time point, and more than 10 sections for each mouse were analyzed. Data are presented as means ± SEs (error bars; *P < 0.05, **P < 0.01, ***P < 0.001)

We have previously demonstrated that *RNF13* expression gradually decreases during skeletal muscle development, and over-expression of *RNF13* inhibits muscle cell proliferation (Zhang et al., 2010). *RNF1*3^*-/-*^ mice display no overt physiological abnormalities during development, as previously described (Zhang et al., 2013b); in the present study, *RNF1*3^*-/-*^ mice were used to assess the significance of RNF13 function in regulating skeletal muscle regeneration. To this end, we established a CTX-induced muscle regeneration model in *RNF13*^*-/-*^ and *RNF13*^+/+^ mice. *RNF13*^*-/-*^ and *RNF13*^+/+^ mice were intramuscularly injected with CTX in the tibialis anterior (TA) muscles and analyzed histologically at 1, 3, 5, and 7 d after injury (Fig. 1A). At 1 d after injury, the muscle cross sections showed that some fibers were damaged and immune cells infiltrated in *RNF13*^*-/-*^ and *RNF13*^+/+^ mice (Fig. 1A). No phenotypic changes were observed in either of the genotypes. At 3 d and 5 d after injury, H&E staining of the injured TA muscles revealed significantly more regenerating fibers characterized by their centrally located nuclei in *RNF13*^*-/-*^ mice than in *RNF13*^+/+^ mice (Fig. 1B). The cross-sectional areas (CSAs) of regenerating muscle fibers at 5 d and 7 d after injury were analyzed and showed that the regenerating muscle fibers in *RNF13*^*-/-*^ mice were larger than those in *RNF13*^+/+^ mice (Fig. 1C and 1D). These results showed that the muscles from *RNF13*^*-/-*^ mice exhibited an enhanced regeneration compared with those from *RNF13*^+/+^ mice after CTX damage.

### Satellite cells proliferate more rapidly in *RNF13*^*-/-*^ mice than in *RNF13*^+/+^ mice

**Figure 2:**
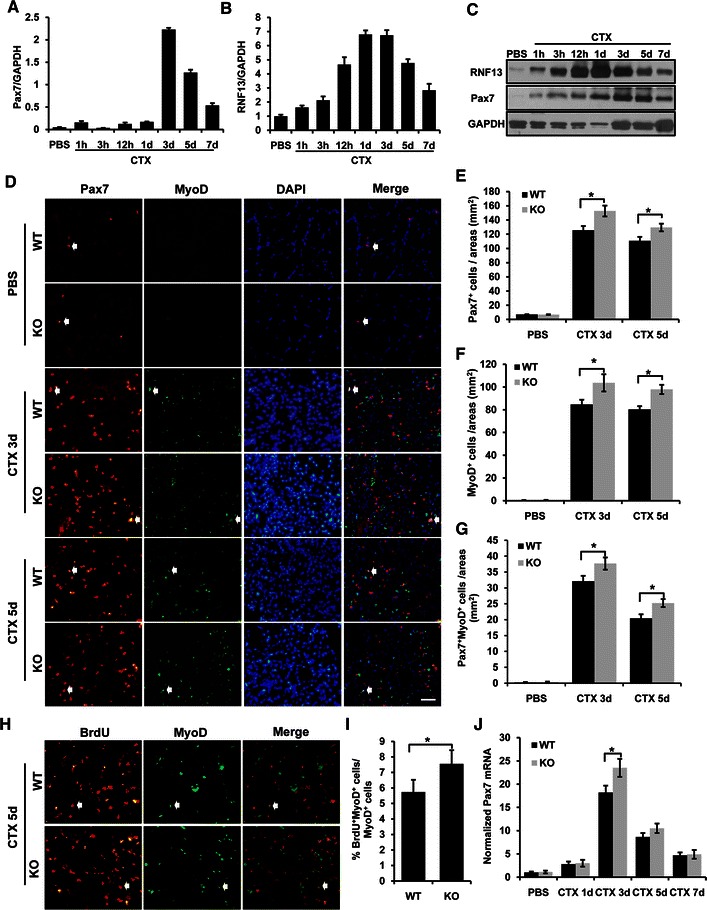
**Satellite cell proliferation is accelerated in*RNF13***^**-/-**^**mice**. (A–C) Pax7 and RNF13 mRNA and protein expression were determined by qRT-PCR and Western blot, respectively, at different time points after damage. (D) Frozen sections of TA muscles from *RNF13*^-/-^ and *RNF13*^+/+^ mice damaged for 3 d or 5 d were immunostained for Pax7 (red), MyoD (green), and nuclei (DAPI; blue). The right panel shows a merged image. (E–G) Pax7^+^, MyoD^+^, and Pax7^+^/MyoD^+^ cells in defined areas were counted. More than 50 sections from five individuals were analyzed. (H) Frozen sections of TA muscles from *RNF13*^-/-^ and *RNF13*^+/+^ mice damaged for 5 d were immunostained for BrdU (red) and MyoD (green). (I) The percentage of BrdU^+^ cells in the MyoD^+^ cell population was determined. More than 50 sections from five individuals were analyzed. (J) Pax7 mRNA levels in uninjured (0 d) and injured muscles at different times after injury (1, 3, 5, and 7 d) from *RNF13*^-/-^ and *RNF13*^+/+^ mice were determined by qRT-PCR. Scale bar = 200 μm. Data are presented as means ± SEs (error bars; *P < 0.05, **P < 0.01, ***P < 0.001)

The improved skeletal muscle regeneration following injury in *RNF13*^*-/-*^ mice prompted us to investigate the stages of skeletal muscle regeneration in which RNF13 is involved. To accomplish this objective, we initially examined the expression pattern of RNF13 during regeneration in wild-type mice. We found that RNF13 expression was remarkably up-regulated immediately after injury and reached the peak at day 1 (Fig. 2A–C). The significant induction of RNF13 expression at an early stage of muscle regeneration demonstrated the function of RNF13 in regulating satellite cell activation and proliferation, which are important for an efficient muscle regeneration. To test this possibility, we examined whether or not satellite cell activation and proliferation are altered in *RNF13*^*-/-*^ mice by immunofluorescence staining for Pax7 and MyoD (Fig. 2D). The transcription factor Pax7 is a marker of quiescent satellite cells as well as activated, proliferating satellite cells during muscle regeneration, whereas MyoD is considered as a marker for activated and proliferating satellite cells. Pax7^+^, MyoD^+^, and Pax7^+^/MyoD^+^ cells were counted and revealed a greater number of activated and proliferating satellite cells in *RNF13*^-/-^ mice at 3 d and 5 d after injury than in *RNF13*^+/+^ mice (Fig. 2E–G). To determine the accelerated proliferation in *RNF13*^-/-^ mice, we performed BrdU and MyoD double staining (Fig. 2H). The statistical analysis results showed that the number of BrdU^+^/MyoD^+^ cells was also increased in *RNF13*^-/-^ mice (Fig. 2I). To further confirm this result, we examined Pax7 expression in muscles at different time points after damage using qRT-PCR and Western blotting analysis (Figs. 2J, S1A, and S1B). The results were consistent with those of immunofluorescence staining, indicating that satellite cells were activated and proliferated more rapidly in *RNF13*^-/-^ mice than in *RNF13*^+/+^ mice.

### Accelerated satellite cell proliferation in *RNF13*^-/-^ mice is mainly non-cell-autonomous

**Figure 3:**
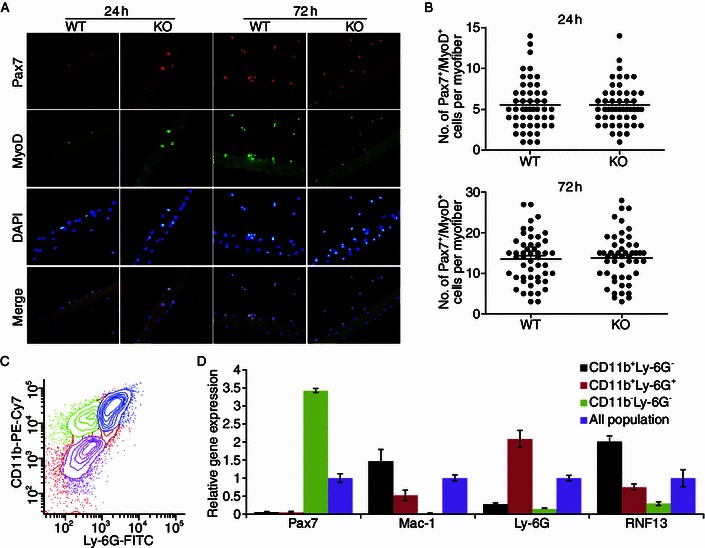
**Accelerated satellite cell activation and proliferation in*RNF13***^***-/-***^**mice is mainly non-cell autonomous**. (A) Single myofibers isolated from *RNF13*^-/-^ and *RNF13*^+/+^ mice were cultured for 24 h or 72 h and stained for Pax7 (red), MyoD (green), and DAPI (blue). The bottom panel shows a merged image. (B) Pax7^+^/MyoD^+^ cells in each individual fiber were counted after 24 h or 72 h of culture. More than 200 fibers from *RNF13*^-/-^ and *RNF13*^+/+^ mice were analyzed. (C) Inflammatory cells were sorted by FACS. CD11b^+^/Ly-6G^+^ cells are considered neutrophils, and CD11b^+^/Ly-6G^-^ cells are considered macrophages. (D) The levels of Mac-1 (macrophage marker), Ly-6G (neutrophil marker), Pax7 (satellite cell marker), and RNF13 mRNA in sorted cells were determined by qRT-PCR. Similar results were obtained in three separate experiments

Considering that satellite cell activation and proliferation are accelerated in *RNF13*^-/-^ mice, we then investigated whether the improvement in satellite cell function is cell autonomous or non-cell autonomous. To test this hypothesis, we assessed the effects of RNF13 on satellite cell activation and proliferation in isolated, cultured single myofibers. In this model, satellite cells initially express Pax7 only; MyoD is subsequently expressed and cells enter the cell cycle. Proliferating cells either down-regulate Pax7, leading to differentiation, or down-regulate MyoD, leading to self-renew (Halevy et al., 2004; Olguin and Olwin, 2004; Zammit et al., 2004). After 24 h or 72 h in culture, the fibers were stained for Pax7 and MyoD (Fig. 3A). Pax7^+^/MyoD^+^ cells were then counted to assess the satellite cell activation and proliferation. We found no difference in the number of Pax7^+^/MyoD^+^ cells between the single myofibers isolated from *RNF13*^-/-^ mice and *RNF13*^+/+^ mice (Fig. 3B). This result suggested that the modulation of satellite cell activation and proliferation by RNF13 ablation is dependent on the niche provided by other cells.

Muscle injury causes an inflammatory immune response in damaged tissues (Tidball, 2005), and inflammatory reaction plays a critical regulatory role in satellite cell activation and proliferation. Considering that RNF13 did not directly affect satellite cell function, we hypothesized that aberrant inflammatory responses contributed to the accelerated regeneration of *RNF13*^-/-^ muscles. In addition, RNF13 expression was induced as early as 1 h after CTX-induced muscle damage; this condition occurred earlier than the upregulation of Pax7 (Fig. 2A–C), suggesting that RNF13 exerts its functions before satellite cell activation. To determine the specific cells contributing to RNF13 induction after damage, we separated the cells in damaged TA muscles by flow cytometry (Fig. 3C). Two main subsets of inflammatory cells in damaged muscles can be distinguished on the basis of Ly-6G and CD11b expressions: CD11b^+^/Ly-6G^+^ cells, which are considered as neutrophils, and CD11b^+^/Ly-6G^-^ cells, which are considered as macrophages. CD11b^-^/Ly-6G^-^ cells, which were also collected, are non-inflammatory cells. To assess the purity of the sorted cells, we assessed the expressions of the macrophage marker Mac-1, the neutrophil marker Ly-6G, and the satellite cell marker Pax7 by qRT-PCR analysis (Fig. 3D). Pax7 was highly expressed in the CD11b^-^/Ly-6G^-^ population, suggesting that this population enriched the satellite cells. The RNF13 expression pattern in these sorted cells was analyzed and the results revealed that RNF13 induced after CTX damage was mainly expressed in inflammatory cells, particularly in macrophages (Fig. 3D). These results indicated that RNF13 regulates satellite cell functions by influencing inflammatory cells. The accelerated satellite cell proliferation observed in *RNF13*^-/-^ mice is mainly non-cell autonomous.

### Loss of RNF13 induces cytokine production without influencing the recruitment of inflammatory cells

**Figure 4:**
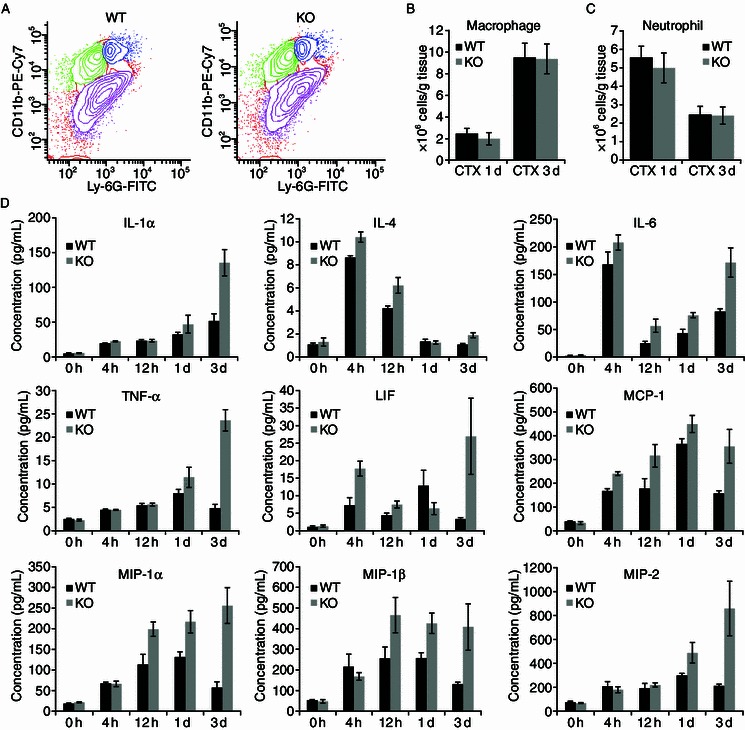
**Loss of RNF13 induces the production of cytokines without influencing the recruitment of inflammatory cells**. (A) FACS analysis of CD11b^+^/Ly-6G^+^ cells (macrophages) and CD11b^+^/Ly-6G^-^ cells (neutrophils) in muscle-derived cells from *RNF13*^-/-^ and *RNF13*^+/+^ mice damaged for 1 d or 3 d. (B and C) The number of intramuscular macrophages and neutrophils at 1 d and 3 d after damage was analyzed. Similar results were obtained in three separate experiments. (D) The concentrations of 18 cytokines and chemokines in damaged TA muscles from *RNF13*^-/-^ and *RNF13*^+/+^ mice were determined at 0, 4, and 12 h, and 1 d and 3 d post-injury using a Luminex multiplex assay. Shown are 9 of 18 cytokines and chemokines whose concentrations in the muscles changed after injury in *RNF13*^-/-^ mice. Data are presented as means ± SEs (error bars; *P < 0.05, **P < 0.01, ***P < 0.001; *n* = 5 mice/time point)

RNF13 is significantly induced in inflammatory cells after injury; thus, RNF13 regulates muscle regeneration primarily by altering muscle inflammation instead of directly affecting satellite cell function. To further confirm this result, we initially investigated whether or not RNF13 deficiency affects inflammatory cell infiltration. We subsequently counted the numbers of intramuscular macrophages and neutrophils in the injured muscles by flow cytometry (Fig. 4A). We found no significant difference in the numbers of intramuscular macrophages or neutrophils between *RNF13*^-/-^ mice and *RNF13*^+/+^ mice at 1 d or 3 d after damage (Fig. 4B and 4C). Therefore, RNF13 deficiency did not influence the recruitment of inflammatory cells in response to acute injury. We then identified the factor(s) in the niche required for the RNF13 activation of satellite cells at the early stage of muscle regeneration. Considering that cytokines and chemokines are important in muscle regeneration, we determined the presence of 18 different cytokines/chemokines secreted in TA muscles at different time points after CTX-induced damage (0, 4, and 12 h, and 1 d and 3 d) by using a Luminex multiplex platform (Table S2). All of the 18 cytokines/chemokines were stimulated after injury, and the concentrations of nine cytokines, particularly IL-4, IL-6, TNF-α, IL-1α, MIP-1α, and MIP-1β, were greater in *RNF13*^-/-^ skeletal muscles than in *RNF13*^+/+^ skeletal muscles (Fig. 4D). These results suggested that RNF13 deficiency regulates muscle regeneration by affecting the concentration of specific cytokines rather than by influencing the accumulation of inflammatory cells.

### RNF13 regulates muscle regeneration by modulating IL-4/IL-6 levels secreted by macrophages

**Figure 5:**
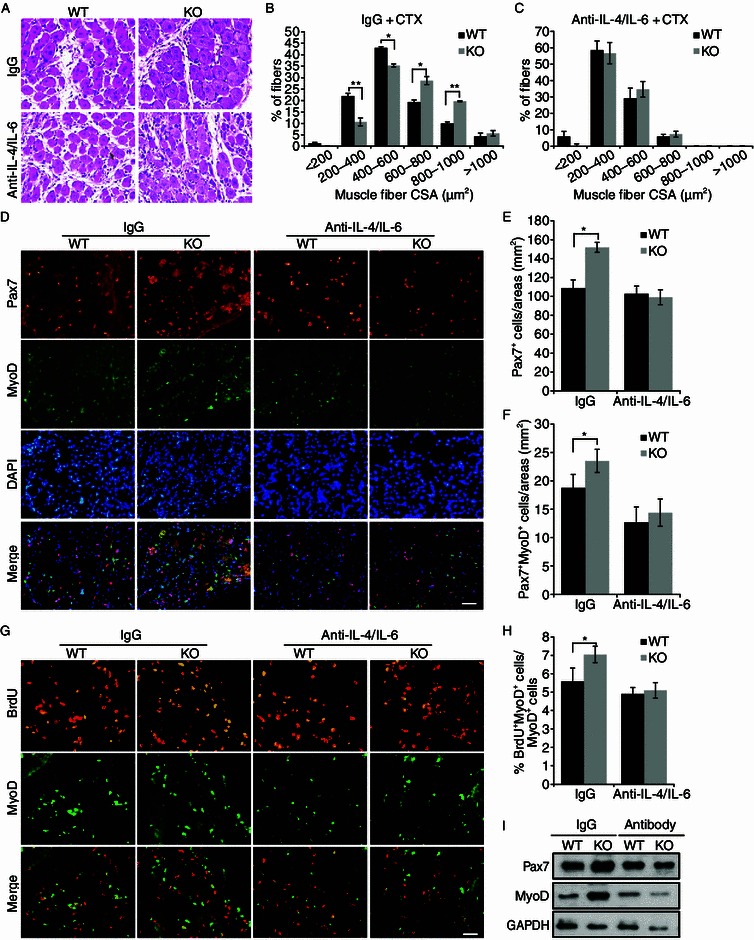
**RNF13 regulates muscle regeneration by enhancing IL-4/IL-6 expression**. (A) Neutralizing antibodies against IL-4 and IL-6 (or control IgG antibodies) were injected into the TA muscles of *RNF13*^+/+^ and *RNF13*^-/-^ mice before CTX damage. Sections from TA muscles damaged for 5 d were stained with H&E. Scale bar = 200 μm. (B and C) The CSAs of regenerating muscle fibers were analyzed in H&E-stained sections. Five pairs of mice were used at each time point, and more than 10 sections from each mouse were analyzed. (D) Frozen sections of TA muscles, injected with anti-IL-4/IL-6 or control antibodies, were immunostained for Pax7 (red), MyoD (green), and nuclei (DAPI; blue). The bottom panel shows a merged image. (E and F) Pax7^+^ and Pax7^+^/MyoD^+^ cells in defined areas were counted. More than 50 sections from five individuals were analyzed. (G) Frozen sections of TA muscles injected with anti-IL-4/IL-6 or control antibodies were immunostained for BrdU (red) and MyoD (green). (H) The percentage of BrdU^+^ cells in the MyoD^+^ cell population was determined. More than 50 sections from five individuals were analyzed. (I) Pax7 and MyoD expression in the damaged muscle injected with anti-IL-4/IL-6 or control antibodies was detected by Western blot. Data are presented as means ± SEs (error bars; *P < 0.05, **P < 0.01, ***P < 0.001)

The concentrations of important cytokines and chemokines increased in the damaged muscles of *RNF13*^-/-^ mice. However, this result fails to clarify whether or not this induction can be accounted for the accelerated muscle regeneration in *RNF13*^-/-^ mice. If this condition is possible, the specific cytokines involved in this process remain unknown. Among the nine cytokines that showed greater changes in *RNF13*^-/-^ mice, IL-4 and IL-6 were remarkably and significantly upregulated within 4 h after muscle damage in *RNF13*^-/-^ mice compared with *RNF13*^+/+^ mice (Fig. 4D). These results suggested that IL-4 and IL-6 are possible mediators of RNF13 function. These results are also consistent with those in previous studies, in which IL-4 and IL-6 are important in skeletal muscle regeneration (Heredia et al., 2013; Zhang et al., 2013a). Given the fact that RNF13 is significantly induced in the macrophages of damaged muscles after injury, we hypothesized that the increased levels of IL-4 and IL-6 in such macrophages from *RNF13*^-/-^ mice were due to the loss of RNF13 function in these macrophages. Thus, we initially examined the expressions of IL-4 and IL-6 in different populations of sorted cells from wild-type mice. These two cytokines were also mainly expressed in the macrophages in the damaged muscles (Fig. S2A) and exhibited the similar expression manner with RNF13. This observation was further supported by the significantly increased mRNA levels of IL-4 and IL-6 in sorted macrophages from the damaged muscles (4 h after injury) of *RNF13*^-/-^ mice compared with *RNF13*^+/+^ mice (Fig. S2B). Finally, we investigated the functional connection between IL-4/IL-6 upregulation and improved muscle regeneration in *RNF13*^-/-^ mice by a neutralization experiment. We injected neutralizing antibodies against IL-4 and IL-6 into the TA muscles of *RNF13*^+/+^ and *RNF13*^-/-^ mice, respectively, before CTX damage. We then analyzed the regeneration after 5 d. Morphological analysis results showed that blocking IL-4 and IL-6 substantially slowed the regeneration process (Fig. 5A). In particular, the analysis results of fiber size showed that the acceleration of muscle regeneration in *RNF13*^-/-^ mice was prevented by blocking IL-4 and IL-6 eliminated (Fig. 5B and 5C; compared with IgG control). Frozen sections were stained concurrently for Pax7, MyoD, and BrdU to evaluate satellite cell activation and proliferation (Fig. 5D and 5G). Consistent with the morphological data, this immunohistochemical analysis showed that the numbers of Pax7^+^/MyoD^+^ cells and BrdU^+^/MyoD^+^ cells in RNF13^-/-^ mice decreased after the antibodies were injected (Fig. 5E, 5F and 5H). These results were also confirmed by detection of Pax7 and MyoD expression in the damaged muscle by Western blotting (Fig. 5I). Collectively, these findings indicate that RNF13 regulates satellite cell function and muscle regeneration by modulating IL-4/IL-6 levels in the satellite cell niche.

## Discussion

This study provided the first evidence showing that RNF13 deficiency promoted skeletal muscle regeneration after CTX-induced muscle damage. In addition, cytokine levels in the damaged TA muscles of *RNF13*^-/-^ mice, particularly those of IL-4 and IL-6, differed from those of *RNF13*^+/+^ mice. These levels further showed that the accelerated muscle regeneration induced by RNF13 deficiency was partially reversed by blocking IL-4/IL-6 function. Taken together, these results indicated that membrane-associated E3 ligase RNF13 regulated muscle regeneration by modulating IL-4/IL-6 levels.

The Ub-proteasome system (UPS) is a major pathway of protein degradation in mammalian cells. UPS also plays a critical role in the breakdown of muscle proteins during regeneration. Furthermore, UPS selectively degrades target proteins following their ubiquitination by different E3 ligases. In addition to functioning in protein degradation, increasing specific E3 ligases have been found to play a significant role in muscle regeneration. The loss of RNF5, a membrane-associated E3 ligase, is reported to lead to delayed muscle regeneration after CTX damage (Delaunay et al., 2008). Cbl-b, another ubiquitin E3 ligase, functions via RANTES production in macrophages by regulating cytotoxic T-cell infiltration into regenerating muscles. The ablation of Cbl-b gene expression delays muscle regeneration following CTX-induced muscle damage (Kohno et al., 2011). Another recent study demonstrated that the E3 ligase TRIM32 is expressed in the skeletal muscle stem cell lineage of adult mice; myogenic differentiation is disrupted and muscle regeneration is delayed in the absence of TRIM32 (Nicklas et al., 2012). TRAF6, also an ubiquitin E3 ligase, positively regulates myogenic differentiation, thereby promoting myogenic differentiation and muscle regeneration via TAK1/p38 MAPK and Akt pathways (Xiao et al., 2012). The current study defined a novel function of the E3 ligase RNF13. In particular, RNF13 regulates muscle regeneration by mediating the inflammation phase. Considering the findings in the present study and previous studies, we found that E3 ligase-mediated protein modification is important in the regulation of skeletal muscle development and regeneration.

The inflammatory response is a coordinated process that should be finely regulated if an efficient regenerative process is initiated; this progression depends on a balance between excessive and insufficient inflammatory activities (Tidball and Villalta, 2010). In particular, functional inflammation requires well-regulated inflammatory cell infiltration and cytokine secretion. For example, CC chemokine ligand 2 (CCL2) regulates inflammatory responses by affecting the mobilization of macrophages from the bone marrow to the blood and from the blood to injured muscles (Lu et al., 2011). In addition, the local expression of insulin-like growth factor 1 (IGF-1) rapidly modulates specific inflammatory factors, thereby accelerating regeneration by influencing cytokine secretion after injury (Pelosi et al., 2007).

In our study, RNF13 deficiency did not influence the infiltration of neutrophils or macrophages into the damaged areas. Thus, cytokine concentrations were surprisingly altered in *RNF13*^-/-^ mice. Among the important cytokines upregulated in *RNF13*^-/-^ mice, IL-4 and IL-6 were involved in RNF13 function. In our previous study, snapin, a member of the SNARE complex, was found as a substrate of RNF13. Snapin, expressed ubiquitously in neuronal and non-neuronal cells, interacts with SNAP23 and likely performs a general function in SNARE-mediated vesicle fusion events in non-neuronal cells (BUXTON, 2003). In addition, a subset of t-SNAREs (syntaxin 4/SNAP23/Munc18c) performs a regulatory function in cytokine secretion in macrophages (Pagan, 2003). Therefore, RNF13 likely regulates cytokine secretion in inflammatory cells by modulating t-SNARE assembly, in the same mechanism by which the neuronal SNARE (Vamp2/SNAP25) assembly in the hippocampus is affected (Zhang et al., 2013b). On the basis of this supposition, we confirmed that syntaxin 4, SNAP23, and snapin were selectively expressed in sorted macrophages and neutrophils from damaged muscles by qRT-PCR (Fig. S3A). Considering that RNF13 regulates the association of neuronal SNARE components (Vamp2, SNAP25, and synaptotagmin 1), we also performed co-immunoprecipitation experiments to investigate whether or not RNF13 depletion affects the SNARE complex (syntaxin4 and SNAP23) assembly in sorted macrophages or in the total damaged muscle. Unexpectedly, RNF13 ablation did not regulate the interaction of syntaxin 4 with SNAP23 and snapin (Fig. S3B). Therefore, RNF13 functions in muscle regeneration are not mediated through snapin, suggesting that another substrate of RNF13 is involved. As such, further studies should be conducted to identify the RNF13 substrate(s) involved in the regulation of satellite cell function via macrophage-secreted IL-4/IL-6 in damaged muscles.

Although our results failed to explain the exact mechanism of RNF13 function in detail, the study demonstrated that RNF13 deficiency promotes cytokine production/secretion. Cytokines are a significant part of the satellite cell niche, regulating satellite cell activation, proliferation, and differentiation. The key to normal muscle regeneration is the maintenance of niche homeostasis in response to alterations induced by various physiological stimuli. A deficiency of RNF13 creates a niche that fosters satellite cell function by enhancing IL-4 and IL-6 activities; as a result, muscle regeneration is accelerated. Our finding that RNF13 deficiency promotes muscle regeneration provides new information to develop strategies for the treatment of muscle diseases. This result also emphasizes the importance of targeting specific inflammatory cell populations to optimize therapeutic approaches.

## Materials and methods

### Mice and skeletal muscle regeneration

The generation of *RNF13*^-/-^ mice has been described previously (Zhang et al., 2013b). *RNF13*^-/-^ and *RNF*13^+/+^ mice were produced by crossing *RNF13*^+/-^ heterozygous mice. The animals were handled and cared for in accordance with the guidelines of the Animal Ethics Committee of Peking Union Medical College. Muscle regeneration was monitored in mice (8–10 weeks old) after inducing muscle injury by injecting 20 μL of 10 μmol/L CTX (Sigma) in phosphate-buffered saline (PBS) into the mid-belly of the right TA muscle; the left TA muscle was injected with PBS (20 μL) as a control. For rescue experiments, IL-4 and IL-6 antibodies (10 μL of 1 μg/μL in PBS; Biolegend) were injected into the TA muscle 2 h before CTX injection.

### Muscle histology and hematoxylin and eosin staining

TA muscles were harvested 1, 3, 5, and 7 d following CTX injury. The muscles were fixed in 4% paraformaldehyde (Sigma) in PBS overnight and paraffin embedded for hematoxylin and eosin (H&E) staining. The proportion of fibers with central nuclei (regenerating fibers) was counted in the injured area, and the CSAs of the fibers were measured using Image Pro Plus 5.1 software (Olympus).

### Immunofluorescence staining of frozen sections

For Pax7 immunostaining, fresh sections were fixed in 4% paraformaldehyde for 20 min, permeabilized with methanol (-20°C) for 6 min, and blocked first with a solution containing 4% bovine serum albumen (BSA; Jackson) in PBS and then with goat anti-mouse AffiniPure Fab fragment (1:100; Jackson) after antigen retrieval with 100 mmol/L sodium citrate. The sections were then incubated overnight at 4°C with an anti-Pax7 antibody (1:20; Developmental Studies Hybridoma Bank). After washing with PBS, Pax7 signals were visualized by incubating with biotin-conjugated goat anti-mouse IgG_1_ (1:1000; Jackson) and Cy3-conjugated streptavidin (Jackson; 1:2500). Nuclei were stained with 4’,6-diamidino-2-phenylindole (DAPI; Invitrogen). For MyoD immunostaining, the fresh sections were fixed in 4% paraformaldehyde for 20 min, permeabilized with 0.2% Triton X-100/PBS (PBST) for 10 min, and blocked by incubating with 5% BSA/6% goat serum/0.2% PBST at room temperature for 2 h. Immunostaining with anti-MyoD antibody (1:50; Santa Cruz) was performed by overnight incubation at 4°C. After washing, immunoreactive proteins were visualized by incubating with fluorescein isothiocyanate (FITC)-conjugated goat anti-rabbit IgG. Nuclei were stained with DAPI (Roche). The Pax7^+^ and MyoD^+^ cells in the sections (*n* = 10) were counted.

### BrdU incorporation assay

For *in vivo* BrdU incorporation assays, 125 mg/kg of BrdU (Sigma) was injected into TA muscles 2 h prior to harvesting. Frozen sections were fixed in 4% paraformaldehyde for 20 min, incubated in 2 mol/L HCl at 37°C for 30 min for DNA denaturation, and then immersed twice in 0.1 mol/L borate buffer (5 min each) to neutralize the acid. After three washes with PBS, the sections were blocked with 5% goat serum for 30 min and then incubated with anti-BrdU (Abcam) at 4°C overnight. The sections were then washed with PBS and incubated with FITC-conjugated goat anti-rat IgG to visualize BrdU signals. BrdU-positive cells were quantified in 60 sections from six mice.

### Quantitative PCR analysis

Total RNA was extracted using the TRIzol reagent (Life Technologies) and reverse transcribed using RevertAid reverse transcriptase (Thermo Scientific). Quantitative reverse transcription-polymerase chain reaction (qRT-PCR) analyses were performed using a Bio-Rad iQ5 Multicolor Real-Time PCR Detection System (Bio-Rad). The primer sequences are listed in Table S1.

### Western blot analysis

Muscle tissues were lysed in a buffer containing 50 mmol/L Tris pH 7.5, 150 mmol/L NaCl, 0.5% Nonidet P-40, and protease and phosphatase inhibitors. Protein lysates were resolved by sodium dodecyl sulfate-polyacrylamide gel electrophoresis and transferred to a polyvinylidene fluoride membrane, followed by immunoblotting with primary antibodies against Pax7 (Developmental Studies Hybridoma Bank), MyoD (BD Biosciences), and glyceraldehyde 3-phosphate dehydrogenase (GAPDH; Millipore). Membranes were washed for 30 min and incubated with horseradish peroxidase-conjugated secondary antibodies (Zhongshanjinqiao Corp.) for 1 h at room temperature and then washed with PBS for 30 min. Each membrane was then placed into a Detection Solution (Thermo), incubated for 1 min at room temperature, and subsequently exposed to X-ray film. Band densities were quantified using Image J software.

### Single fiber isolation and culture

Single myofibers were isolated from the gastrocnemius muscles of eight-week-old mice by collagenase I (Sigma, C-0130) digestion (Rosenblatt et al., 1995). Each gastrocnemius muscle was harvested and incubated in 3 mL of 0.2% collagenase I in Dulbecco’s modified Eagle’s medium (DMEM; without serum) in a shaking water bath at 35°C for 60–90 min. Digestion was complete when the muscle looked less defined and slightly swollen, with hair-like single fibers appearing to come away from the edge of the muscle. The muscles were placed in a Petri dish, and myofibers were isolated under a microscope. Single fibers were placed in six-well plates pre-coated with Matrigel (1:3; Becton Dickinson) and allowed to attach for 3 min. Then, 2 mL of fiber medium consisting of DMEM supplemented with 10% horse serum, 0.5% chick embryo extract, and 1% antibiotic/antimycotic was added. The fibers were cultured for 24 h or 72 h at 37°C/5% CO_2_, fixed with 4% paraformaldehyde and stained for Pax7 and MyoD, as described above. The numbers of Pax7^+^/MyoD^+^ cells determined from counts of at least 100 single fibers were used for statistical analyses.

### Isolation of macrophages and neutrophils by cell sorting

Flow cytometry was performed using muscle single-cell suspensions. TA muscles, damaged for 1 d or 3 d, were harvested from mice; washed in PBS; minced in 2 mL of digestion solution containing 0.2% collagenase II (Sigma), 2.4 U/mL of dispase (Sigma), and 2.5 mmol/L CaCl_2_; and incubated at 37°C with shaking for 60–90 min. Digestion was stopped by adding an equal volume of 10% FBS in PBS, after which the top liquid layer was filtered through a 40-μm nylon mesh to remove particulates, and centrifuged at 350 *g* for 3 min. The cells were washed twice with PBS and then suspended in PBS. These mononuclear cells were stained with CD11b-PE-CY7 (1:500; BD Biosciences, 561098) and Ly-6G-FITC (1:1000; BD Biosciences, 561105). The cells were sorted using a BD FACS Aria II fluorescence-activated cell sorter. CD11b^+^/Ly-6G^+^ cells were defined as neutrophils and CD11b^+^/Ly-6G^-^ cells were defined as macrophages.

### Cytokine detection

TA muscles from *RNF13*^*-/-*^ and *RNF13*^*+/+*^ mice were harvested at different time points after damage (0, 4 h, and 12 h, and 1 d and 3 d). Muscle tissues were lysed in a buffer containing 50 mmol/L Tris pH 7.5, 150 mmol/L NaCl, 0.5% Nonidet P-40, and protease and phosphatase inhibitors. The concentration of 18 cytokines and chemokines in the damaged muscles was detected using MILLIPLEX MAP Multiplex Assay Kits, according to the manufacturer’s instructions (Millipore).

### Statistical analysis

Results are presented as means ± SEs. The statistical significance of differences between two means was calculated using Student’s *t*-test. The probability level accepted for significance was *P* < 0.05.

## Electronic supplementary material

Below is the link to the electronic supplementary material.

Supplementary material 1 (PDF 311 kb)

## Abbreviations

CSA: cross-sectional areas
CTX: cardiotoxin
IL-4/6: interleukin 4/6
MyoD: myogenic differentiation antigen
Pax7: paired-box transcription factor
RNF13: RING finger protein 13
SNARE: soluble N-ethylmaleinide-sensitive fusion protein attachment protein receptor
TA: tibialis anterior
UPS: Ub-proteasome system
