# Supplementary material for: Accelerated regeneration of the skeletal muscle in RNF13-knockout mice is mediated by macrophage-secreted IL-4/IL-6
Source: Protein Cell. 2014 Feb 22;5(3):235–47. doi: 10.1007/s13238-014-0025-4 (PMC3967074; doi:10.1007/s13238-014-0025-4)
Supplement: Supplementary file 1 — Supplementary material 1 (PDF 311 kb) [file 13238_2014_25_MOESM1_ESM.pdf]

SUPPLEMENTAL FIGURES

Supplemental Table1 Primers for qRT-PCR

Gene name	primers
Pax7	F: 5’ -CCGTGTTTCTCATGGTTGTG –3’<br>R: 5’- GAGCACTCGGCTAATCGAAC-3’
RNF13	F: 5’-CACAGAGAGCAGGATACAAAGCAGCCATAG-3’<br>R: 5’-GTGGCCCCCTTTTTCATATGTG-3’
GAPDH	F: 5'-TGGAGAAACCTGCCAAGTATGA-3'<br>R: 5'- CTGTTGAAGTCGCAGGAGACA-3'
Mac-1	F: 5'-CTGCCTCAGGGATCCGTAAAG-3'<br>R: 5'-CCTCTGCCTCAGGAATGACATC-3'
Ly-6G	F: 5'-TGGACTCTCACAGAAGCAAAG-3'<br>R: 5'-GCAGAGGTCTTCCTTCCAACA-3'
IL-4	F: 5’-ATGTGCCAAACGTCCTCACAG-3’<br>R: 5’- AAGCACCTTGGAAGCCCTACA
IL-6	F: 5’-TAGTCCTTCCTACCCCAATTTCC-3’<br>R: 5’-TTGGTCCTTAGCCACTCCTTC-3’
MCP-1	F: 5’-TCTCACTGAAGCCAGCTCTCTCT-3’<br>R: 5’-CAGGCCCAGAAGCATGACA-3’
TNF-α	F: 5’- AAATGGCCTCCCTCTCATCAG-3’<br>R: 5’- TCCACTTGGTGGTTTGCTACG-3’
Syntaxin4	F:5'-ACAGGACCCACGAGTTGA-3'<br>R: 5'-GCCATAGTCTGCCGAATT-3'
SNAP23	F:5'-GCATCTGCCCTTGTAATA-3'<br>R:5'-GGCTGCTCCTGTAGTTTG-3'
snapin	F: 5'-TTAATGCCAGGCGACGAG-3'<br>R: 5'-CTTGGAGAACCAGGAGGG-3'

Supplemental Table2 Eighteen cytokines detected with the Luminex Kit

**GM-CSF**	**IFN-ϒ**	**IL-1α**	**IL-1β**	**IL-4**	**IL-6**
**LIF**	**IL-10**	**IL-12(p40)**	**IL-12 (p70)**	**IL-17**	**MCP-1**
**LIX**	**MIP-1α**	**MIP-1β**	**MIP-2**	**TNF-α**	**RANTES**

Supplemental Fig.1 Pax7 and MyoD expression is more in the RNF13$^{-/-}$ mice than in RNF13$^{+/+}$ mice on protein level

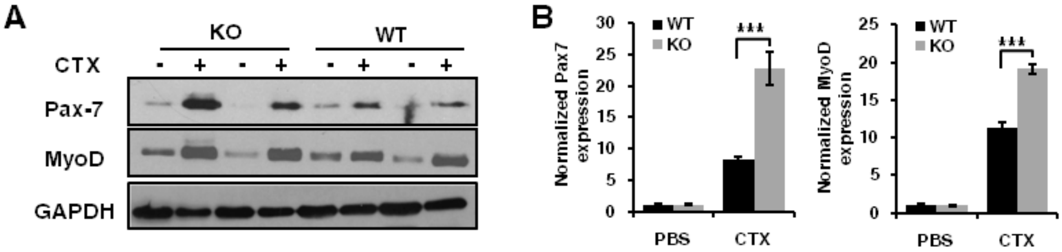

(A) Pax7 and MyoD protein level in uninjured (0d) and injured muscles (3d) was checked by western blot. (B) The expression of Pax7 and MyoD was normalized to GAPDH. Error bars represent means ±SEs (*P<0.05, **P<0.01, ***P<0.001).

Supplemental Fig.2 IL-4 and IL-6 were mainly produced by macrophages in damaged muscles

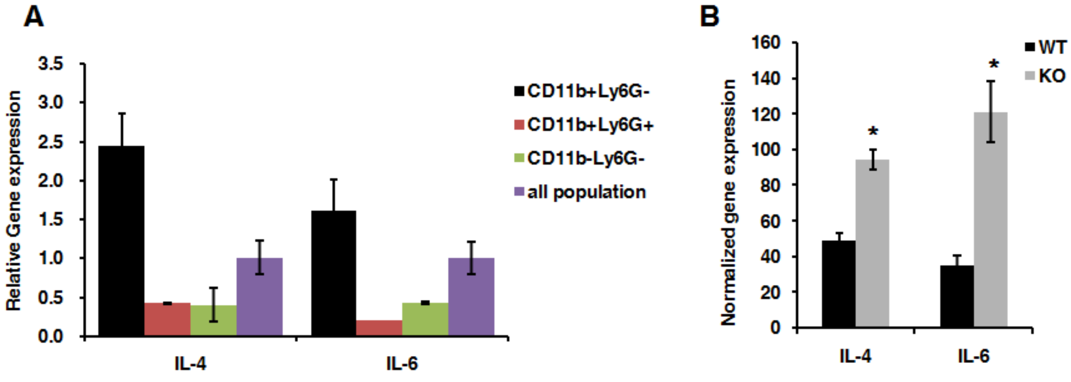

(A) Gene expression of IL-4 and IL-6 in sorted macrophages, neutrophils and other cells from wild type mice damaged for 4hrs was detected by qRT-PCR. Similar results were obtained in three separate experiments. (B) Gene expression of IL-4 and IL-6 in sorted macrophages from *RNF13*$^{-/-}$ and *RNF13*$^{+/+}$ mice damaged for 4hrs was detected by qRT-PCR.

Supplemental Fig.3 RNF13does not regulate the interaction of syntaxin4 with SNAP23 and snapin

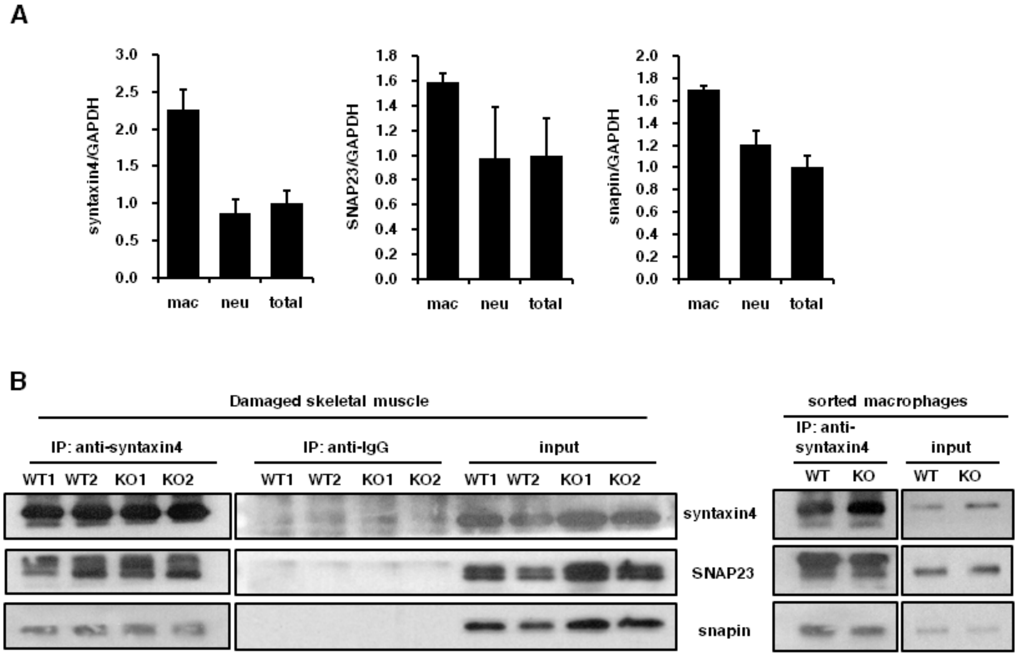

(A) Gene expression of syntaxin4, SNAP23 and snapin in sorted macrophages and neutrophils was detected by qRT-PCR. (B) Sorted macrophages lysate and total damaged muscle lysate immunoprecipitation by syntaxin4 antibody to detect the related SNARE complex proteins, SNAP23 and snapin. Similar results were obtained in three separate experiments.
